# Attribution of country level foodborne disease to food group and food types in three African countries: Conclusions from a structured expert judgment study

**DOI:** 10.1371/journal.pntd.0010663

**Published:** 2022-09-12

**Authors:** Amanda C. Sapp, Mirna P. Amaya, Arie H. Havelaar, Gabriela F. Nane

**Affiliations:** 1 Emerging Pathogens Institute, Food Systems Institute, Animal Sciences Department, University of Florida, Gainesville, Florida, United States of America; 2 Delft Institute of Applied Mathematics, Delft University of Technology, Delft, The Netherlands; George Washington University Medical Center, UNITED STATES

## Abstract

**Background:**

According to the World Health Organization, 600 million cases of foodborne disease occurred in 2010. To inform risk management strategies aimed at reducing this burden, attribution to specific foods is necessary.

**Objective:**

We present attribution estimates for foodborne pathogens (*Campylobacter* spp., enterotoxigenic *Escherichia coli* (ETEC), Shiga-toxin producing *E*. *coli*, nontyphoidal *Salmonella enterica*, *Cryptosporidium* spp., *Brucella* spp., and *Mycobacterium bovis*) in three African countries (Burkina Faso, Ethiopia, Rwanda) to support risk assessment and cost-benefit analysis in three projects aimed at increasing safety of beef, dairy, poultry meat and vegetables in these countries.

**Methods:**

We used the same methodology as the World Health Organization, i.e., Structured Expert Judgment according to Cooke’s Classical Model, using three different panels for the three countries. Experts were interviewed remotely and completed calibration questions during the interview without access to any resources. They then completed target questions after the interview, using resources as considered necessary. Expert data were validated using two objective measures, calibration score or statistical accuracy, and information score. Performance-based weights were derived from the two measures to aggregate experts’ distributions into a so-called decision maker. The analysis was made using Excalibur software, and resulting distributions were normalized using Monte Carlo simulation.

**Results:**

Individual experts’ uncertainty assessments resulted in modest statistical accuracy and high information scores, suggesting overconfident assessments. Nevertheless, the optimized item-weighted decision maker was statistically accurate and informative. While there is no evidence that animal pathogenic ETEC strains are infectious to humans, a sizeable proportion of ETEC illness was attributed to animal source foods as experts considered contamination of food products by infected food handlers can occur at any step in the food chain. For all pathogens, a major share of the burden was attributed to food groups of interest. Within food groups, the highest attribution was to products consumed raw, but processed products were also considered important sources of infection.

**Conclusions:**

Cooke’s Classical Model with performance-based weighting provided robust uncertainty estimates of the attribution of foodborne disease in three African countries. Attribution estimates will be combined with country-level estimates of the burden of foodborne disease to inform decision making by national authorities.

## Author summary

The World Health Organization has presented estimates of the burden of foodborne disease and attribution percentages of zoonotic foodborne pathogens to food groups (e.g., beef, vegetables). To inform food safety decision making at the national level, these estimates should be combined, extended to pathogens with human reservoirs and provided at a more detailed level (e.g., drinking raw milk as one food product in the dairy group). We present a Structured Expert Elicitation study using the same protocol as the World Health Organization (Cooke’s Classical Model) to attribute foodborne disease of selected pathogens (*Campylobacter* spp., enterotoxigenic *Escherichia coli*, Shiga-toxin producing *E*. *coli*, nontyphoidal *Salmonella enterica*, *Cryptosporidium* spp., *Brucella* spp., *Mycobacterium bovis*) to different food types and food products in the beef, dairy, poultry and vegetable groups in three African countries (Burkina Faso, Ethiopia, and Rwanda). Individual experts’ uncertainty assessments resulted in modest statistical accuracy and high information scores, that is concentrated in small ranges, suggesting overconfident assessments. Nevertheless, the optimized item-weighted decision maker was statistically accurate and informative. Cooke’s Classical Model with performance-based weighting provided robust uncertainty estimates of the attribution of foodborne disease in three African countries.

## Introduction

Infections of humans by enteric pathogens can occur through various transmission routes including food, water, air, soil, human-to-human contact, and animal-to-human contact. Due to these various pathways, burden estimates of foodborne disease are challenging. The Foodborne Disease Burden Epidemiology Reference Group (FERG) was established by the World Health Organization (WHO) in 2007 with the aim to develop the first worldwide estimates of the burden of foodborne disease on a sub-regional level. Subregions were based on the official grouping of WHO Member States. FERG further subdivided each region into subregions based on child and adult mortality as described by [1]: very low child and adult mortality (stratum A), low child mortality and very low adult mortality (stratum B), low child mortality and high adult mortality (stratum C), high child and adult mortality (stratum D), and high child mortality and very high adult mortality (stratum E). Burkina Faso was assigned to the Africa Region (AFR), stratum D (AFRD) and Ethiopia and Rwanda to AFR, stratum E (AFRE). FERG has used Structured Expert Judgment according to Cooke’s Classical Model [2] to quantify the relative impact of foodborne disease compared to other transmission routes [3]. Factors considered by the experts included the epidemiology of the foodborne hazards (microorganism or chemical) causing the disease, seasonality, patterns of food consumption, geographic regions, as well as the ecology of the hazards. According to the World Health Organization [4], 600 million cases of foodborne disease were estimated to have occurred globally in 2010. Of these, enteric pathogens accounted for 550 million cases. The African sub-regions D and E were observed to have the highest burden of foodborne disease among all sub-regions analyzed: 13,000 and 12,000 Disability Adjusted Life Years (DALYs) per 100,000 population, respectively.

Global attribution estimates of foodborne disease to specific food groups were first published in 2017 for a limited number of zoonotic pathogens [5]. Such estimates are not yet available for many pathogens that have human reservoirs, such as *Shigella* spp. and *Escherichia coli* pathotypes. Building on these estimates, Li *et al*., estimated the global and sub-regional burden of pathogens in animal source foods [6]. Estimates of disease burden attributed to specific food groups, food types and food products are not yet available at country level.

We present a study on food attribution to support the objectives of three projects aiming to improve food safety in three African countries:

1. *The Assessment and Management of Risk from Non- typhoidal Salmonella*, *Diarrheagenic Escherichia coli and Campylobacter in Raw Beef and Dairy in Ethiopia* (TARTARE) [7];
2. *Urban Food Markets in Africa* (Pull-Push) [8] and
3. *Rwanda Enhancement for Enabling Policy Support to the Dairy Sector* (RD) [9].

All projects had a similar need for foodborne disease burden data attributed to specific food products to support food safety decision making. To complement attribution estimates available from FERG, we conducted three country-specific Structures Expert Judgment studies, which are reported in this paper. Separate papers will report burden estimates, based on FERG data and the attribution estimates presented elsewhere [10,11].

## Methods

### Ethical statement

Approvals were obtained from the University of Florida Institutional Review Board (IRB 201901947 -Ethiopia, 202001259-Burkina Faso, 202001265-Rwanda), from the International Livestock Research Institute Institutional Research Ethics Committee (ILRI-IREC2020-06, Ethiopia and Burkina Faso) and from the University of Rwanda College of Medicine and Health Sciences Institutional Review Board (129/CHMS IRB/2020). Written consent was received from all participating experts.

### Countries and scope

**Fig 1:**
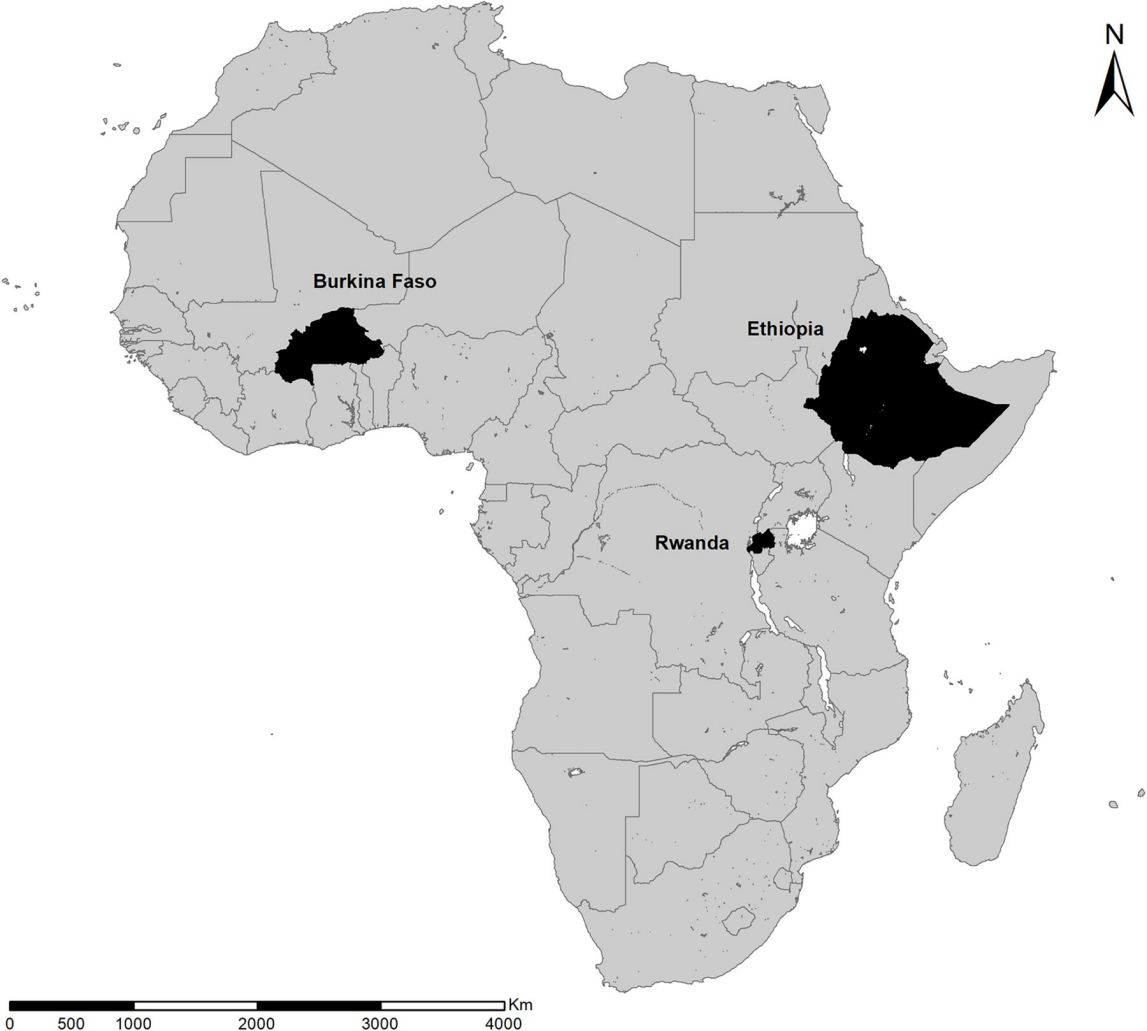
Geographic location of study countries in Africa. Source: https://hub.worldpop.org/geodata/summary?id=29691.

The three projects consider food safety in Burkina Faso, Ethiopia and Rwanda (Fig 1). Based on infant and adult mortality, Burkina Faso (2017 population 15,605,210) was assigned to the AFRD subregion and Ethiopia (2017 population 106,399,926) and Rwanda (2017 population 11,980,960) to the AFRE sub-region by FERG [12]. Table 1 presents the objectives and scope of the three projects. Different food groups and different hazards were chosen for consideration in these projects.

**Table 1:** Scope and objectives of three food safety projects in Africa and associated food classification schemes and hazards.

Project short name	Study objective(s) and study period	Food groups	Food types	Food products	Hazards of interest*
**TARTARE** Project lead: The Ohio State University Local partners: Ethiopia Public Health Institute, Addis Ababa University, Haramaya University	To increase equitable consumption of a safe, affordable, and nutritious diet by reducing morbidity and mortality from foodborne disease using Ethiopia as a model. 2019–2023	Beef	Beef types - Red meat (lean or fatty) - Offal (dulet)	Red meat products - Consumed raw - Jerky (qwanta) - Semi-cooked (lebleb) - Cooked	*Campylobacter*, *Salmonella* and STEC
		Dairy	Dairy types - Milk from cattle - Milk from other animals	Cattle milk products - Consumed raw - Consumed pasteurized and boiled - Fermented by traditional processes (ergo) - Cottage cheese by traditional processes (ayib) - Other types of cattle milks	*Campylobacter*, *Salmonella* and STEC
**Pull Push** Project lead: International Livestock Research Insitute Local partners: Addis Ababa University, Haramaya University	To reduce the burden of foodborne disease by building capacity of food chain actors and regulators to cost-effectively mitigate important food safety risks in the poultry and vegetable value chain by incentives of harnessing consumer demand for food safety, using in Ethiopia and Burkina Faso as a model. 2019–2023	Poultry	Poultry types - Chicken meat	Chicken meat products - At home bought raw (includes live chicken slaughtered at home) - At home bought processed - Outside of the home	*Campylobacter* and *Salmonella*
		Vegetables	Vegetable types - Tomatoes - Leafy greens - Cabbage - Green peppers - Onion - Other vegetable types	Tomato products - Consumed raw - Consumed semi-cooked	Salmonella and ETEC
**Rwanda Dairy** Project lead: University of Florida Local partner: University of Rwanda	To analyze and develop policy options to promote the stimulation of increased production and marketing of processed milk and milk products for both the domestic and export markets in Rwanda. 2019–2021	Dairy	Dairy types - Milk from cattle - Milk from other animals.	Cattle milk products - Consumed raw - Fermented by traditional processes (e.g., ikivugoto) -—Fermented by industrial processes - Heat treated - Other	*Campylobacter*, *Salmonella Brucella*, *Cryptosporidium* and *M*. *bovis*

Enterotoxigenic *Escherichia coli* (ETEC), Shiga-toxin producing *Escherichia coli* (STEC)

Hazards (in this context defined as a biological agent which may cause illness in humans) were selected for each country separately based on the goals for the individual projects as shown in Table 1. The selection of foods and hazards to be included was made jointly by project partners while designing the studies and was informed by the results from the global burden of foodborne disease study [4] and specific concerns in the target countries, as voiced by local partners. We designed a hierarchical scheme to classify foods at three levels. The first level was based on FERG and included the food groups “Beef, Ruminants’ meat, Dairy, Poultry, Vegetables, Fruits and nuts, Grains and beans, Oils and sugars, and Other foods”. Guided by the goals of the projects and input from experts in each country, food groups were subdivided in food types. For example, food types in the Dairy category included “Milk from cattle and Milk from other species” while food types in the Vegetables category included “Tomatoes, Leafy greens, Cabbage, Green pepper, Onions, and Other vegetables”. Only food types of interest for the three projects were further subdivided in food products, representing specific consumer products. These products were defined on a country basis as necessary. For example, in Ethiopia, the Milk from cattle food type included the food products “Raw, Fermented traditional (ergo), Cottage cheese traditional (ayib), Heat treated, and Other products” while in Rwanda, this food type included the food products “Raw, Fermented traditional, Fermented industrial, Heat treated and Other products”. Within each category, items were defined to be mutually exclusive and exhaustive (i.e., attribution estimates should sum to 100%). Experts were asked to provide conditional assessments for food group domains. For example, of all cases of foodborne disease due to *Campylobacter*, what percentage of these cases is attributable to consumption of Red meat? And for all cases attributed to Red meat, what proportion is attributable to Raw meat? For enterotoxigenic *Escherichia coli* (ETEC), we also elicited attribution to food groups while FERG results [5] were used for other hazards.

### Point of attribution

The point of attribution for food groups, food types and food products were based on FERG who have defined this as “the point where the hazards entered the place where the foods are prepared for final consumption” [3]. For example, a person may become ill from eating a fresh green salad that was contaminated by a knife previously used to cut raw chicken. If the chicken had been contaminated with pathogens prior to entering the food preparation area, we would attribute the illness to the chicken, not the lettuce.

### Cooke’s classical model

The Classical Model for Structured Expert Judgment is a method to elicit, validate and aggregate expert opinion for uncertainty quantification [2,13]. The experts are asked to quantify their uncertainty about quantities of interest, by providing subjective assessments for percentiles of the distributions. Usually the 5^th^, 50^th^ and the 95^th^ percentiles, denoting the best estimate (50^th^ percentile), along with a credible interval, provided by the 5^th^ and 95^th^ percentiles. The validation is enabled by calibration questions, whose realizations are not known to experts but are known to analysts. The objective evaluation of experts’ assessments is enabled by a calibration and an information score, which are derived from the calibration questions. The calibration score measures the statistical accuracy of expert’s assessments, that is, relative to expert’s assessments, how discrepant the distribution of the realizations is to the expected relative frequencies of the realizations. We expect 5% of the realizations to fall below expert’s 5^th^ percentiles and 5% of the realizations to fall above the 95^th^ percentiles, and 90% of the realizations to fall between the 5^th^ and the 95^th^ percentiles, with 45% above and 45% below the median. The information score denotes how concentrated expert’s assessments are with respect to a background measure. A combined score, obtained by multiplying the calibration score with the information score is used to evaluate the overall performance of experts and it is also used to compute performance-based weights. Experts’ three elicited quantiles are used to construct so-called minimum informative distributions, that is, distributions which don’t assume any parametric form, nor other information apart from the three quantiles. The Classical Model enables the weighted combination of these distributions using various weights, which result in corresponding Decision Makers (DMs). Performance-based weights which result from experts’ normalized combined score lead to a Performance-Based Decision Maker. Experts’ distributions for the calibration questions can be aggregated as well, the same performance-based weights and therefore the Performance-Based Decision Maker can be evaluated using the calibration and information score, just as for any expert. Various performance-based weights can be considered in aggregating distributions, such as global weights, which are computed using all calibration questions, and item weights, which are derived for each question. Alternatively, optimized Decision Makers search for subsets of experts whose aggregated distributions lead to the best performing Performance-Based Decision Makers in terms of both the calibration and information score. Finally, equal weights can also be considered, and the performance of all resulting Decision Makers can be compared using their calibration and information score.

We note that unlike data-driven models, which rely on large number of observations, expert judgment methods typically employ, due to practical constraints, a limited number of experts. The Classical Model accepts a minimum number of 4 experts and advises for at least 6 experts to participate in a study [13]. A robustness analysis provides insights into how robust DMs performance is with respect to each expert or calibration question.

### Expert identification, invitation, selection, and panels

**Fig 2:**
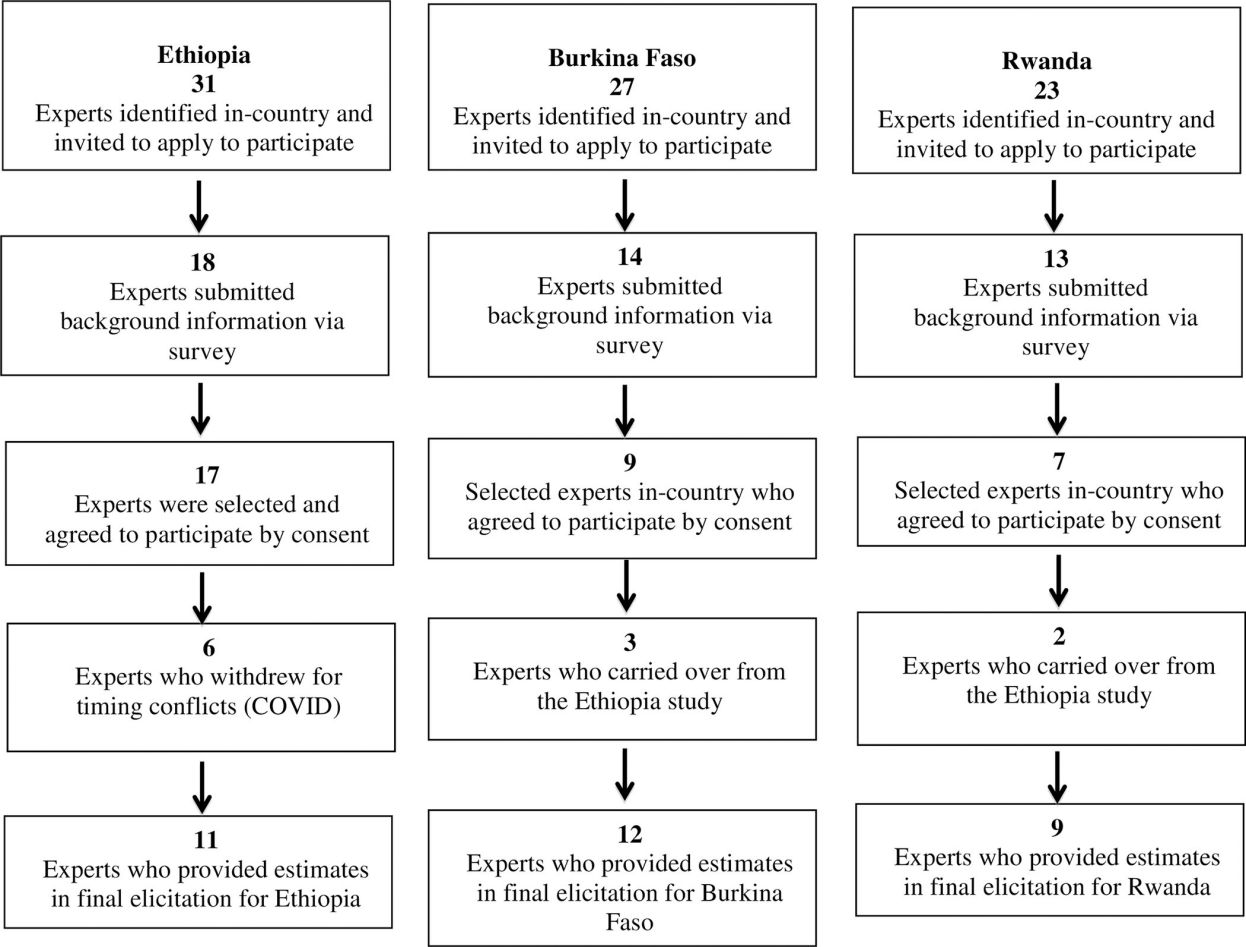
Expert identification, selection and enrollment for three African Structured Expert Judgment studies.

Identification of experts was carried out by the study team for each project within each country. In total, 31 experts were identified in Ethiopia, 27 in Burkina Faso and 23 in Rwanda. Of these, 11, 12 and 9 participated in the study (Fig 2). The identified experts were sent a formal letter of invitation by email to participate in the study and, if interested, complete a Qualtrics (Qualtrics, Provo, UT) survey using a link provided in the invitation letter. The survey consisted of questions pertaining to their working knowledge and experience.

Experts with appropriate domain knowledge were selected by project principal investigators and project managers based upon the survey. Here, appropriate knowledge was defined as expertise in one or more areas of diarrheal disease in humans, zoonoses, microbial food safety, water and sanitation or veterinary public health. Selected experts were sent an email informing them of their selection into the study for their appropriate country, along with biographies of the interviewers. Selected experts were asked to return a signed consent form and were also asked to recommend colleagues not yet invited as potential participants. In addition, experts who served in one of the three studies, and who had working knowledge in one or more other study countries, were invited to the subsequent studies where appropriate.

Of the 31 identified experts in Ethiopia, 18 submitted detailed background information, 17 were selected and enrolled by consent. Of these, 6 experts withdrew consent due to time conflicts or due to COVID-19 when invited for an interview. In total, 11 experts provided estimates in Ethiopia. For Burkina Faso 27 experts were identified, 14 experts submitted detailed background information, 9 experts were selected and enrolled by consent, and 3 experts carried over from the Ethiopia study. Overall, 12 experts provided estimates in Burkina Faso. For the Rwanda study, 23 experts were identified, 13 submitted detailed background information, 7 were selected and enrolled by consent and 2 experts carried over from the Ethiopia study. For Rwanda, 9 experts provided estimates (Fig 2). Experts were not remunerated for their inputs.

Expert’s education is summarized in Table 2. In all countries, veterinary medicine (including veterinary public health) was the most frequent education, followed by biological sciences (including microbiology) in Burkina Faso and Rwanda but not in Ethiopia. Other backgrounds included agricultural sciences, environmental and food sciences, medical doctor, and public health (including epidemiology). All experts in Burkina Faso held PhD degrees, in Ethiopia there were 8 PhDs and in Rwanda 7. In all countries, most experts were affiliated with universities (6, 8, 7 in Burkina Faso, Ethiopia and Rwanda, respectively), followed by research and development institutes (6, 2 and 2). In Ethiopia, 1 expert worked for a government institute.

**Table 2:** Expert educational backgrounds (highest degree obtained).

Education	Burkina Faso	Ethiopia	Rwanda
Agricultural Sciences	1		1
Biological Sciences	5		3
Environmental and Food Sciences	1	2	2
Medical Doctor		1	
Public health		3	
Veterinary medicine	5	5	3

### Expert training

A background document was prepared for each country separately and provided in advance to the experts. This document included a description of the concepts of uncertainty, variability, and probabilities, as well as an explanation of the calibration and target questions, a definition of the point of attribution, published background information and estimates on the epidemiology of the pathogens of interest. In addition, the background document detailed the procedure such as interview duration, agenda items, and an explanation on how to use Zoom software to participate in the interview and how to complete the survey instrument. All experts completed a training video on providing quantitative estimates under uncertainty (Training video for expert elicitation—YouTube).

### Expert interviews

Interviews were held via the Zoom platform and had a duration of 60–90 minutes. Interviews covered welcome and introduction to the study and an interactive discussion covering the concept of uncertainty. Subsequently, experts provided assessments for the calibration questions during the interview without access to any resources. Then, interviewers walked through an example target question with the experts. After the interview, experts were allotted two weeks to research and complete answers to the target questions, which were returned by email to the interviewer. Experts were encouraged to use resources as they considered necessary while evaluating target questions.

### Calibration questions

All panels included the same series of 10 calibration questions that covered three general themes regarding the continent of Africa. These questions were chosen to reflect the broad domains of the study, i.e., infectious diseases and food and referred to data from Africa, but not from any of the countries to which the study applied. We did not the experts to know any of the answers exactly, but they should be able to produce reasonable estimates based on their general background knowledge. If they were less knowledgeable in a specific domain, we expected them to acknowledge this by providing wider uncertainty intervals. Interviewers presented the questions during the interview and requested answers based solely on current expert domain knowledge. All questions were completed during the interview and answers were sent to the interviewer by email before the interview concluded. Table 3 provides some example questions, while S1 Table provides the full set of calibration questions.

**Table 3:** Sample calibration questions presented to experts in the three African countries.

Themes	Question
**Theme 1: The West Africa Ebola epidemic from 2013 to 2016.**	What was the total number of cases reported during the 2013–2016 Ebola outbreak from the three most affected countries Sierra Leone, Liberia and Guinea combined?
**Theme 2: Diarrhea in Mali**	What is the percent of diarrheal deaths in all children under the age of five years old in Mali for 2012 to 2013?
**Theme 3: Food production in Uganda**	What is the average amount of animal proteins which a person used in Uganda in grams per day from 2005 to 2013?

If an expert participated in more than one study, their calibration assessments were re-used for all subsequent studies, reducing the burden to experts for participating in more than one study.

### Target questions

**Fig 3:**
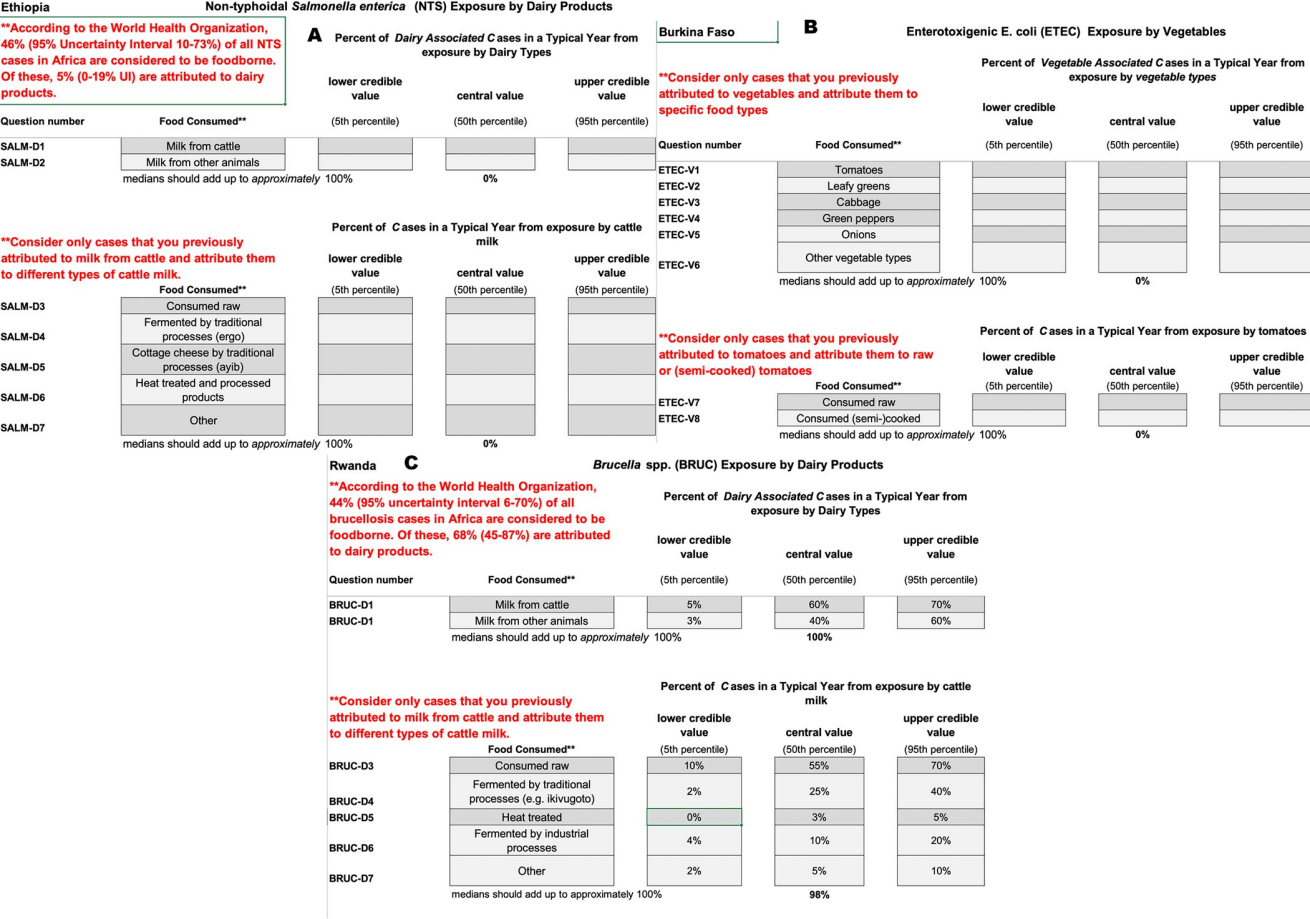
Example target question used in Ethiopia to attribute the burden of foodborne disease to food groups (ETEC only), food types or food products.

Target questions consisted of combinations of hazards with food groups and food types for attribution by country. For each combination, experts were asked to attribute the incidence of foodborne disease in a typical year. Target questions differed between countries, reflecting the food-hazard combinations detailed in Table 1. Estimates were collected by an Excel spreadsheet where each individual tab represented a specific pathogen in one country. An example set of questions and the Excel instrument used to collect the data is shown in Fig 3. The instrument included two quality checks to support the experts to provide valid estimates. For each food group, type or product, the expected sum of medians was provided. The experts were instructed that the sum of the medians should be close to, but not necessarily exactly 100%. An indicator (****) was shown next to each set of uncertainty estimates. The indicator would disappear if all three estimates for a target question were completed and 5^th^ percentile < 50^th^ percentile < 95^th^ percentile. Strictly increasing percentiles are a theoretical requirement of the Classical Model when eliciting continuous distributions. All experts were allotted two weeks to return their target estimates, but exceptions were granted as necessary.

### Data analysis

Individual expert assessments were imported into Excalibur software [14] for each country separately and evaluated using their calibration and information score. The theoretical background is described in [13]. Experts’ assessments were combined using performance-based weights, both optimized and non-optimized, along with equal weights. The resulting Decision Makers’ performance was evaluated and the Decision Maker with the highest combined score selected.

We performed an expert and item-wise robustness analysis on the Ethiopia expert results. For expert-wise robustness analysis, each individual expert was removed from the pool of experts and the impact on the resulting item weighted optimized Decision Maker was assessed. Similarly, each calibration question (item) was removed, and the item re-weighted optimized Decision Maker’s performance were evaluated.

The item weighted optimized Decision Maker’s distributions were used to obtain uncertainty estimates for the attribution of foodborne disease in the three countries. The attribution in food groups is based on FERG, while attribution to food type and food products is based on expert judgment elicited for these three studies specifically.

100 percentiles from the Decision Maker’s uncertainty distribution were exported from Excalibur for each target question in each of the three countries. These percentiles were imported in R software version 4.1.0 [15] and 10,000 observations were jointly sampled from each target variable whose distribution is characterized by these percentiles. A normalization procedure was applied to tuples of jointly selected observations. For example, each tuple with joint samples of food groups for a given hazard was normalized to sum to 100%. The procedure ensured that, as appropriate, the resulting sample mean attribution estimates for all hazards in all food groups, types and products summed up to 100% and provided 10,000 normalized samples of the uncertainty distribution. Normalized samples were thus obtained for each corresponding target question. While the estimates for food types or food products were elicited conditional on corresponding food groups or food types, the mean and 95% credible interval of unconditional attribution estimates were calculated for further combination with foodborne disease burden estimates.

Treemaps were constructed using the *treemapify* package in R [16] to provide a visual summary of the mean attribution results per hazard and per country. For completeness of the treemaps, mean food group attribution results from FERG [5] were included as appropriate.

## Results

**Fig 4:**
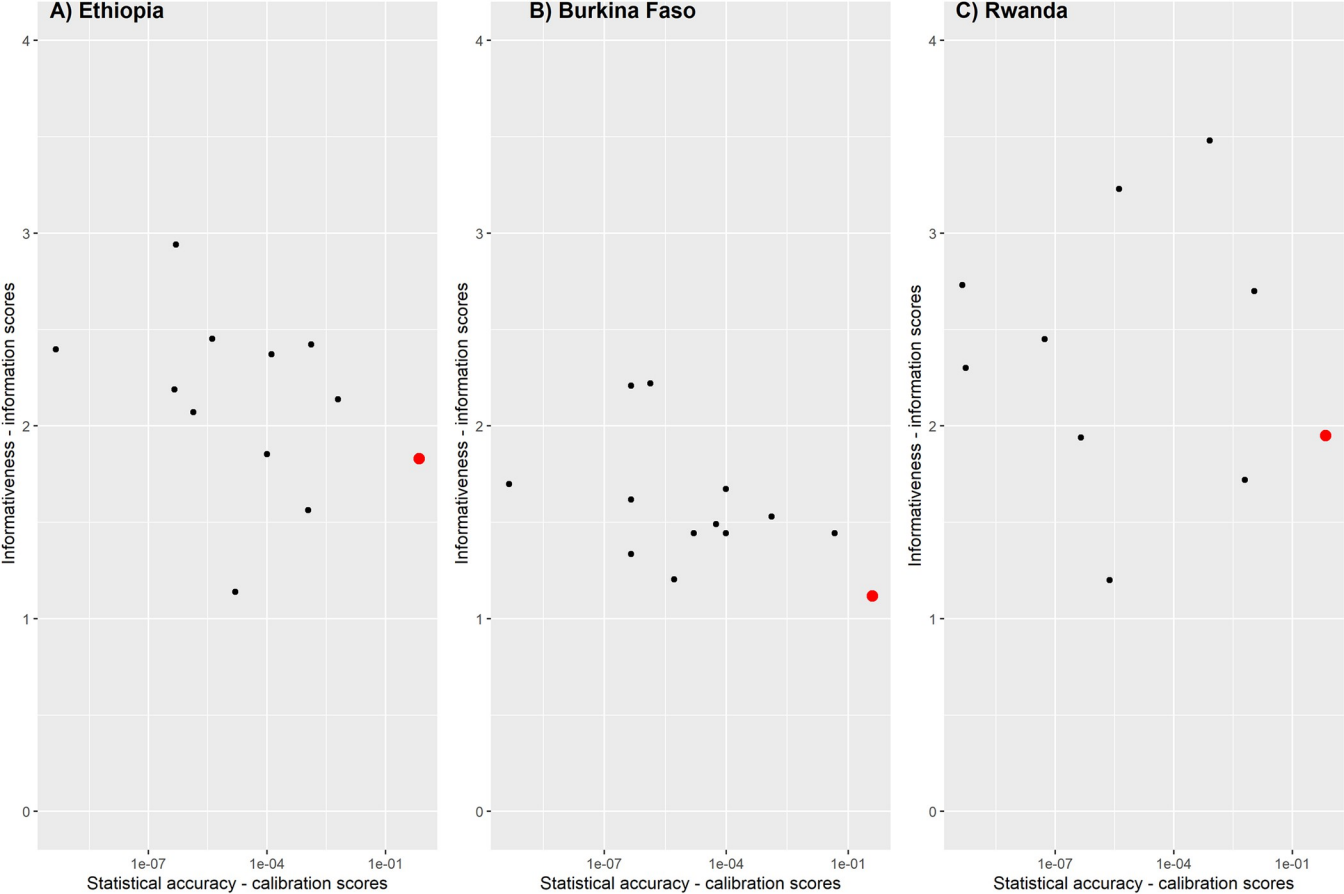
Calibration score (x-axis) by information score (y-axis) for A) Ethiopia, B) Burkina Faso and C) Rwanda. Black dots depict individual experts in the study while red triangles represent the item weights optimized Decision Maker.

The calibration and information scores of the item weights optimized Decision Maker are plotted along with individual expert’s scores in Fig 4. Calibration results were relatively similar in all three countries (Fig 4). No single expert met the threshold of a calibration score greater than 0.05, while information scores were quite high in the range of 1 to 3.5. The information score of experts in Burkina Faso was on average lower than in the other two countries. The Optimized Item Weighted Decision Maker performed best in all three countries. In all countries Decision Maker’s outperformed individual experts in terms of calibration score, which did not occur at a loss of much information. In Ethiopia, the Decision Maker’s calibration score was 0.7, displaying a significant improvement in statistical accuracy, with an information score of 1.8. For Burkina Faso, a calibration score of 0.4 and an information score of 1.1 were obtained. For Rwanda a calibration score of 0.7 and an information score of 2.0 were obtained. All Decision Makers were exceeding the threshold of 0.05. Having information scores higher than 1 implies that the Decision Makers were also informative relative to the uniform background.

Robustness analysis per expert and question revealed that the results were robust with respect to the pool of experts in each country and with respect to the calibration questions. Removing individual experts or questions led to only slight changes in the performance of the Decision Maker’s.

Tables 4–8 present the attribution estimates per country. About ¾ of the foodborne burden by ETEC in Ethiopia was attributed to the food groups of interest (beef, dairy, poultry, and vegetables) (Table 4). In Burkina Faso, about 40% of the foodborne burden of ETEC was attributed to the food groups of interest (poultry and vegetables) (Table 6). Attribution of ETEC to dairy was lower in Burkina Faso (13%, Table 5) than in Ethiopia (24%, Table 4).

**Table 4:** Attribution of foodborne disease due to enterotoxigenic *Escherichia coli* in Ethiopia to food groups.

Food groups	Attribution estimates
Beef	0.13 (0.00, 0.49)^^^
Ruminants’ meat	0.09 (0.00, 0.44)
Dairy	0.24 (0.00, 0.55)
Poultry	0.21 (0.01, 0.52)
Vegetables	0.19 (0.02, 0.56)
Fruits and nuts	0.07 (0.00, 0.42)
Grains and beans	0.03 (0.00, 0.21)
Oils and sugars	0.03 (0.00, 0.18)
Other foods	0.02 (0.00, 0.31)

^^^ Mean (95% uncertainty interval); attribution estimates are conditional on the proportion of disease attributed to food

**Table 5:** Attribution of foodborne disease due to four selected hazards in Ethiopia to food types and food products.

Food groups	Food types	Food products	Attribution estimates
***Campylobacter* spp.**			
Dairy	*Milk from cattle*		*0*.*70 (0*.*06*, *1*.*00)*^*^*^
		Raw	0.36 (0.00, 0.74)
		Fermented traditional (ergo)	0.16 (0.00, 0.50)
		Cottage cheese traditional (ayib)	0.11 (0.00, 0.42)
		Heat treated	0.06 (0.00, 0.34)
		Other products	0.01 (0.00, 0.11)
	*Milk from other animal species*		*0*.*30 (0*.*00*, *0*.*93)*
Beef	*Red meat*		*0*.*60 (0*.*02*, *1*.*00)*^*^*^
		Consumed raw	0.28 (0.00, 0.65)
		Jerky (qwanta)	0.08 (0.00, 0.33)
		Semi-cooked (lebleb)	0.19 (0.00, 0.49)
		Cooked	0.05 (0.00, 0.27)
Poultry	*Chicken meat*		*1*.*00*
		At home, bought raw	0.56 (0.03, 0.90)
		At home bought processed	0.25 (0.00, 0.69)
		Outside of home	0.19 (0.00, 0.60)
**Enterotoxigenic *Escherichia coli***			
Vegetables	*Tomatoes*		*0*.*33 (0*.*01*, *0*.*65)*
		Tomatoes consumed raw	*0*.*22 (0*.*00*, *0*.*49)*
		Tomatoes consumed semi-cooked	*0*.*11 (0*.*00*, *0*.*29)*
	*Leafy greens*		*0*.*21 (0*.*00*, *0*.*61)*
	*Cabbage*		*0*.*17 (0*.*00*, *0*.*59)*
	*Green pepper*		*0*.*16 (0*.*00*, *0*.*55)*
	*Onions*		*0*.*13 (0*.*00*, *0*.*54)*
	*Other vegetables*		*0*.*02 (0*.*00*, *0*.*3)*
**Non-typhoidal *Salmonella enterica***			
Dairy	*Milk from cattle*		*0*.*65 (0*.*12*, *1*.*00)* ^*^*^
		Raw	0.29 (0.02, 0.65)
		Fermented traditional (ergo)	0.18 (0.00, 0.51)
		Cottage cheese traditional (ayib)	0.10 (0.00, 0.39)
		Heat treated	0.06 (0.00, 0.36)
		Other products	0.01 (0.00, 0.10)
	*Milk from other animal species*		*0*.*35 (0*.*00*, *0*.*88)*
Beef	*Red meat*		*0*.*63 (0*.*07*, *0*.*97)*^*^*^
		Consumed raw	0.28 (0.00, 0.59)
		Jerky (qwanta)	0.09 (0.00, 0.33)
		Semi-cooked (lebleb)	0.21 (0.00, 0.47)
		Cooked	0.05 (0.00, 0.28)
	*Offall*		*0*.*36 (0*.*03*, *0*.*92)*
Poultry	*Chicken meat*		*1*.*00*
		At home, bought raw	0.56 (0.07, 0.88)
		At home bought processed	0.25 (0.01, 0.64)
		Outside of home	0.19 (0.00, 0.56)
Vegetables	*Tomatoes*		*0*.*32 (0*.*02*, *0*.*73)*
		Tomatoes consumed raw	*0*.*22 (0*.*01*, *0*.*58)*
		Tomatoes consumed semi-cooked	*0*.*10 (0*.*00*, *0*.*37)*
	*Leafy greens*		0.21 (0.01, 0.61)
	*Cabbage*		0.17 (0.00, 0.59)
	*Green pepper*		0.16 (0.00, 0.55)
	*Onions*		0.13 (0.00, 0.54)
	*Other vegetables*		0.02 (0.00, 0.30)
**Shiga- toxin producing *Escherichia coli***			
Dairy	*Milk from cattle*		*0*.*71 (0*.*20*, *1*.*0)*
		Raw	0.34 (0.04, 0.65)
		Fermented traditional (ergo)	0.18 (0.00, 0.42)
		Cottage cheese traditional (ayib)	0.12 (0.00, 0.34)
		Heat treated	0.06 (0.00, 0.34)
		Other products	0.01 (0.00, 0.11)
	*Milk from other animal species*		*0*.*29 (0*.*00*, *0*.*80)*
Beef	*Red meat*		*0*.*60 (0*.*07*, *1*.*0)*^*^*^
		Consumed raw	0.31 (0.00, 0.66)
		Jerky (qwanta)	0.08 (0.00, 0.27)
		Semi-cooked (lebleb)	0.19 (0.00, 0.46)
		Cooked	0.02 (0.00, 0.20)
	*Offall*		*0*.*40 (0*.*00*, *0*.*93)*

^^^ Mean (95% uncertainty interval); attribution estimates are conditional on the proportion of disease attributed to the relevant food group or type

**Table 6:** Attribution of foodborne disease due to enterotoxigenic *Escherichia coli* in Burkina Faso to FERG food groups.

Food groups	Attribution estimates
Beef	0.13 (0.00, 0.47)^^^
Ruminants’ meat	0.08 (0.00, 0.44)
Dairy	0.13 (0.00, 0.44)
Poultry	0.22 (0.02, 0.56)
Vegetables	0.19 (0.02, 0.53)
Fruits and nuts	0.11 (0.00, 0.45)
Grains and beans	0.03 (0.00, 0.21)
Oils and sugars	0.03 (0.00, 0.25)
Other foods	0.07 (0.00, 0.38)

^^^ Mean (95% uncertainty interval)

**Table 7:** Attribution of foodborne disease in Burkina Faso to food types, and food products.

Food groups	Food types	Food products	Attribution estimates
***Campylobacter* spp.**			
Poultry	*Chicken meat*		*1*.*00*
		At home, bought raw	0.20 (0.00, 0.51)^^^
		At home bought processed	0.32 (0.20, 0.64)
		Outside of home	0.48 (0.11, 0.80)
**Enterotoxigenic *Escherichia coli***			
Vegetables	*Tomatoes*		*0*.*31 (0*.*05*, *0*.*63)*
		Tomatoes consumed raw	0.21 (0.01, 0.49)
		Tomatoes consumed semi-cooked	0.1 (0.00, 0.34)
	*Leafy greens*		*0*.*24 (0*.*02*, *0*.*58)*
	*Cabbage*		*0*.*11 (0*.*00*, *0*.*36)*
	*Green pepper*		*0*.*07 (0*.*00*, *0*.*27)*
	*Onions*		*0*.*12 (0*.*00*, *0*.*4)*
	*Other vegetables*		*0*.*14 (0*.*00*, *0*.*5)*
**Non-typhoidal *Salmonella enterica***			
Poultry	*Chicken meat*		*1*.*00*
		At home, bought raw	0.20 (0.00, 0.52) ^^^
		At home bought processed	0.30 (0.02, 0.60)
		Outside of home	0.50 (0.12, 0.80)
Vegetables	*Tomatoes*		*0*.*33 (0*.*030*, *0*.*64)*
		Tomatoes consumed raw	0.21 (0.00, 0.48)
		Consumed semi-cooked	0.12 (0.00, 0.35)
	*Leafy greens*		*0*.*25 (0*.*02*, *0*.*60)*
	*Cabbage*		*0*.*14 (0*.*02*, *0*.*49)*
	*Green pepper*		*0*.*06 (0*.*00*, *0*.*42)*
	*Onions*		*0*.*08 (0*.*00*, *0*.*27)*
	*Other vegetables*		*0*.*11 (0*.*00*, *0*.*48)*

^^^ Mean (95% uncertainty interval); attribution estimates are conditional on the proportion of disease attributed to the relevant food group

**Table 8:** Attribution of foodborne disease due to five dairy-associated hazards in Rwanda to dairy food types, and food products.

Food groups	Food types	Food products	Attribution estimates
***Campylobacter* spp.**			
	*Milk from cattle*		*0*.*89 (0*.*50–1*.*00)*^^^
		Raw	0.33 (0.00–0.68)
		Fermented traditional	0.19 (0.00–0.56)
		Fermented industrial	0.22 (0.00–0.59)
		Heat treated	0.07 (0.00–0.45)
		Other products	0.08 (0.00–0.43)
	*Milk from other animal species*		*0*.*11 (0*.*00–0*.*50)*
**Non-typhoidal *Salmonella enterica***			
	*Milk from cattle*		*0*.*94 (0*.*50–1*.*00)*
		Raw	0.46 (0.12–0.74)
		Fermented traditional	0.22 (0.01–0.49)
		Fermented industrial	0.15 (0.01–0.42)
		Heat treated	0.06 (0.00–0.43)
		Other products	0.05 (0.00–0.35)
	*Milk from other animal species*		*0*.*06 (0*.*00–0*.*50)*
***Cryptosporidium* spp.**			
	*Milk from cattle*		*0*.*89 (0*.*50–1*.*00)*
		Raw	0.43 (0.05–0.79)
		Fermented traditional	0.23 (0.00–0.57)
		Fermented industrial	0.10 (0.00–0.38)
		Heat treated	0.05 (0.00–0.41)
		Other products	0.08 (0.00–0.34)
	*Milk from other animal species*		*0*.*11 (0*.*00–0*.*50)*
***Brucella* spp.**			
	*Milk from cattle*		*0*.*93 (0*.*49–1*.*00)*
		Raw	0.61 (0.19–0.90)
		Fermented traditional	0.19 (0.00–0.48)
		Fermented industrial	0.06 (0.00–0.32)
		Heat treated	0.04 (0.00–0.42)
		Other products	0.03 (0.00–0.19)
	*Milk from other animal species*		*0*.*07 (0*.*00–0*.*51)*
***Mycobacterium bovis***			
	*Milk from cattle*		*0*.*94 (0*.*50–1*.*00)*
		Raw	0.63 (0.19–0.91)
		Fermented traditional	0.16 (0.01–0.50)
		Fermented industrial	0.06 (0.00–0.34)
		Heat treated	0.05 (0.00–0.43)
		Other products	0.04 (0.00–0.20)
	*Milk from other animal species*		*0*.*06 (0*.*50–1*.*00)*

^^^ Mean (95% uncertainty interval); attribution estimates are conditional on the proportion of disease attributed to the relevant food group

In Ethiopia, about 70 percent of the burden of *Campylobacter*, *Salmonella* and Shiga-toxin producing *Escherichia coli* (STEC) from dairy was attributed to milk from cattle (Table 5) and about ⅓ of the burden of dairy was attributed to consumption of raw cattle milk. About 60% of the burden of these pathogens in beef was attributed to red meat and about 30% to beef consumed raw. For poultry, 100% of the burden was attributed to chicken meat by the study team because meat from other poultry species is consumed very little, if at all. Of the burden of *Campylobacter* and *Salmonella* from poultry meat, 56% was attributed to chicken bought raw and prepared at home. A higher fraction of the burden of *Salmonella* was attributed to tomatoes than of ETEC (0.32, 0.06 respectively) which translated to a higher burden attributed to raw tomatoes (0.22, 0.04 respectively).

The attribution of the burden from vegetables to *Salmonella* and ETEC in Burkina Faso was similar to Ethiopia (Table 7). However, about half of the burden of *Campylobacter* and *Salmonella* from poultry meat was attributed to chicken meat consumed out of the home.

For Rwanda, most of the burden of dairy for all pathogens was attributed to cattle milk (~90 percent, Table 8). Note that the burden of *M*. *bovis* was attributed 100% to dairy by the study team as this pathogen is considered to be exclusively transmitted through milk [3,5]. The attribution to raw milk varied by pathogen with the highest proportions attributed for *Brucella* and *Cryptosporidium* (61% and 63%, respectively) and the lowest for *Campylobacter* (0.33). A substantial proportion of the burden from dairy was also attributed to traditionally fermented cattle milk products (~ 20%, Table 8) and even industrially fermented products (0.06–0.20).

More detailed data including those used to create figures are provided in S2 Table.

**Fig 5:**
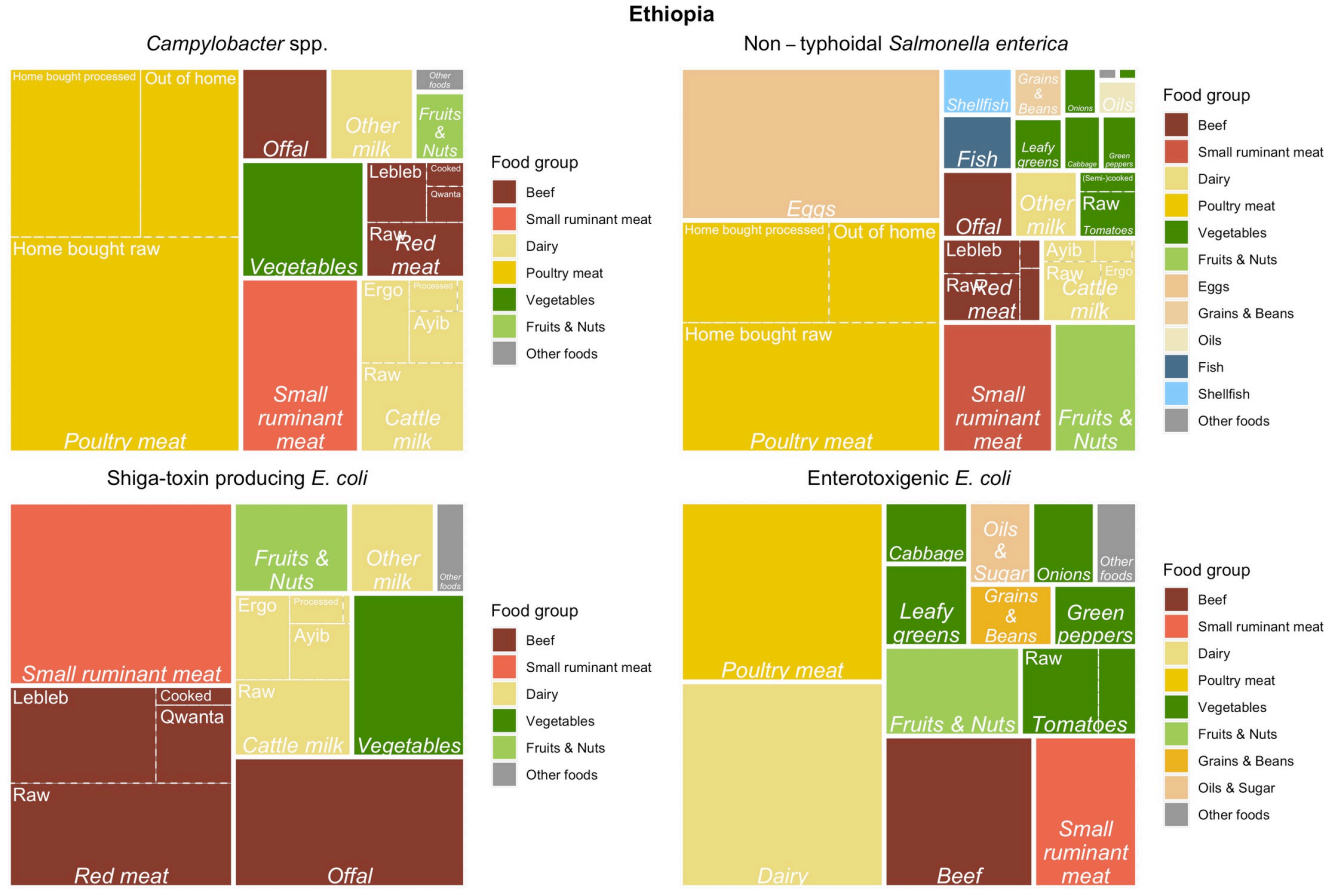
Treemap of mean estimates of the proportion of illnesses caused by *Campylobacter* spp., non-typhoidal *Salmonella enterica*, Shiga toxin-producing *Escherichia coli* and enterotoxigenic *Escherichia coli* through food group/type/product exposure pathways for Ethiopia.

**Fig 6:**
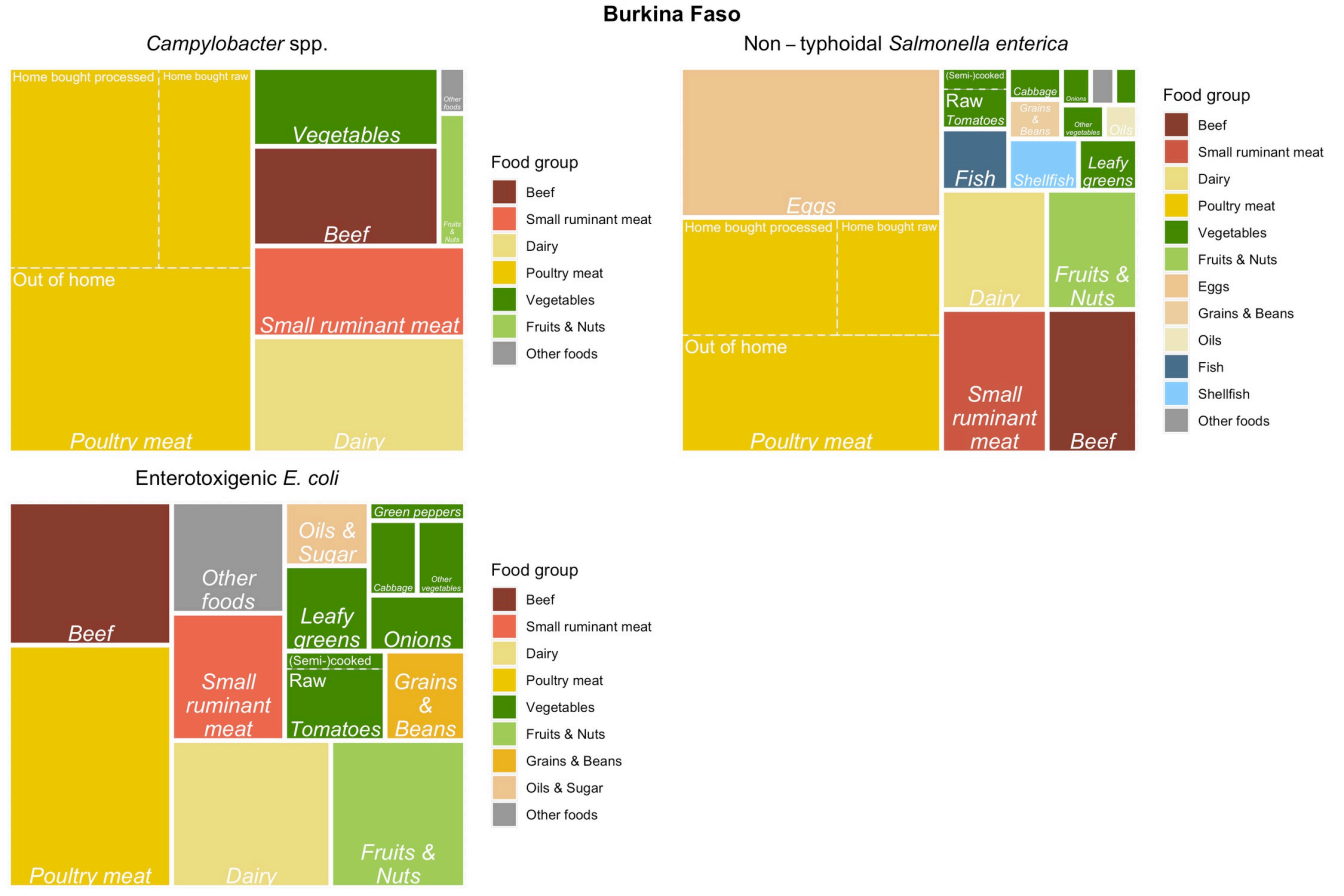
Treemap of mean estimates of the proportion of illnesses caused by *Campylobacter* spp., non-typhoidal *Salmonella enterica* and enterotoxigenic *Escherichia coli* through food group/type/product exposure pathways for Burkina Faso.

**Fig 7:**
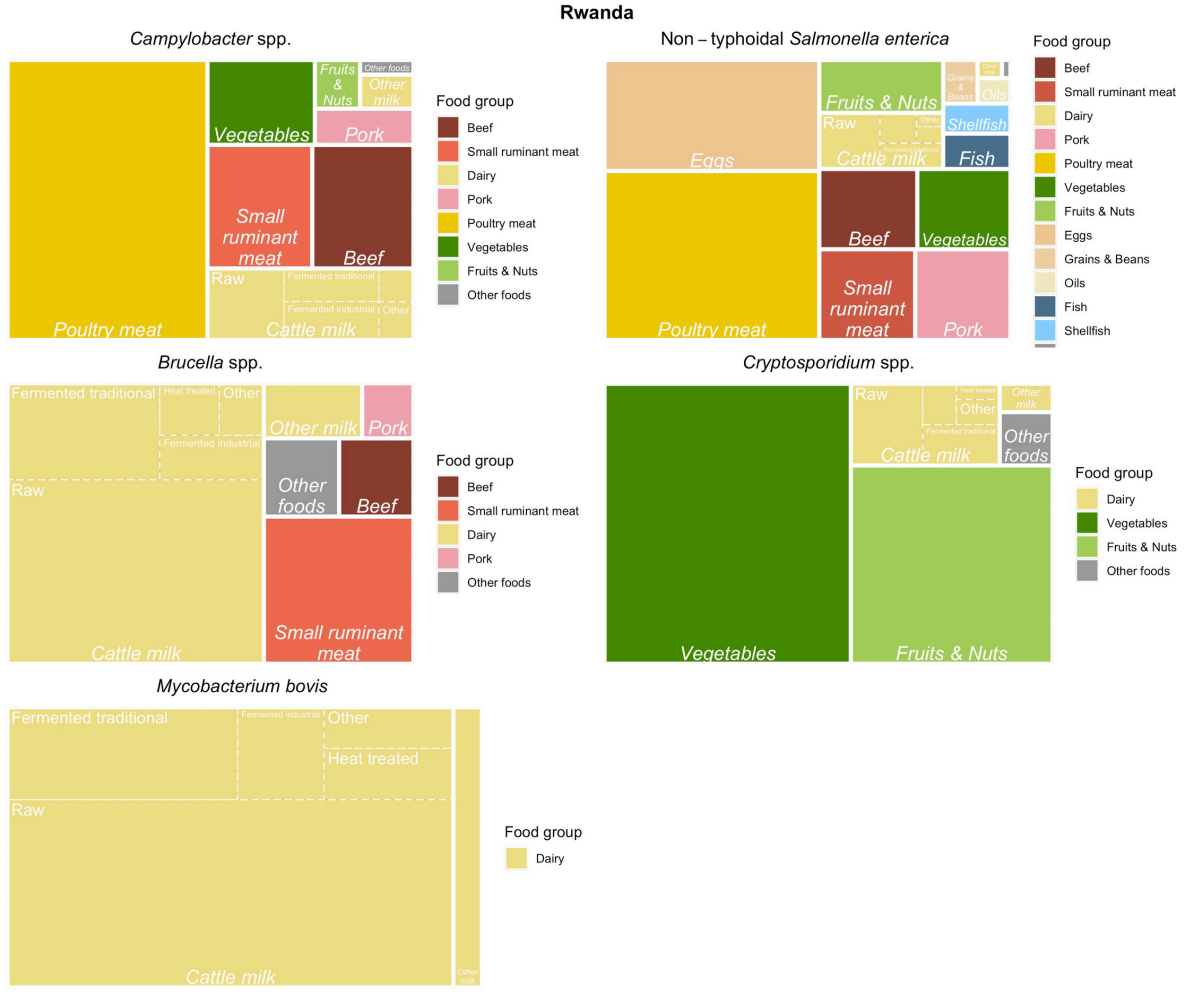
Treemaps of mean estimates of the proportion of illnesses caused by *Campylobacter* spp., non-typhoidal *Salmonella enterica*, *Brucella* spp., *Cryptosporidium* spp. and *Mycobacterium bovis* through food group/type/product exposure pathways for Rwanda.

Tree maps (Figs 5–7) graphically show the results of the experts’ assessments, combined with food group attribution from FERG [5] or this study (for ETEC). Treemaps display hierarchical data as a set of nested rectangles, where the surface area is subdivided according to the proportion of disease attributed to all food groups, food types and food products included in the study. In our case, the surface area of each treemap represents all foodborne disease in a country by a hazard. The surface area is first subdivided according to the attribution to food groups. Food groups are color coded as indicated in the legend to the right of the figure. For example, 68% of all cases of illness by *Brucella* spp. in Rwanda were attributed to dairy (light goldenrod). Second, the surface area of dairy is further subdivided to represent the contribution of different dairy types, represented by italic font. For example, of the 68% *Brucella* spp. cases in Rwanda attributed to dairy, 63% were attributed to milk from cattle. Third, the surface area of milk from cattle is subdivided in five dairy products, represented by regular font. Of the 63% of *Brucella* spp. cases in Rwanda attributed to milk from cattle, 39% were attributed to raw milk consumption.

## Discussion

Individual experts in the three countries did not provide statistically accurate assessments, as all calibration scores were lower than the significance level of 0.05. In turn, their assessments were very informative, with half of the experts’ information scores being higher than 2. This suggests that experts in the study were overconfident–they provided narrow uncertainty margins for the calibration questions which did not include the realizations at the expected relative frequency. Despite having a relatively low number of experts providing assessments, the item-weighted optimized Decision Maker’s in all three countries were statistically accurate. Moreover, the significantly improved statistical accuracy did not come at the cost of low informativeness as all Decision Maker’s information scores were above 1. These results show the power of performance-based weighting and suggest that the range of estimates provided by the experts as a group did represent the realizations to the calibration questions at the expected relative frequencies.

Even though dedicated training sessions were offered to the experts before the interviews, these apparently were not sufficient to prevent overconfident assessments. These results are in contrast with an earlier study in the US, where a large proportion of the experts individually produced well calibrated assessments [17]. An important difference between these two studies was that the US study was performed as an in-person meeting, while the current study was conducted through remote communication. Nonetheless, it needs to be emphasized that empirical evidence suggest that a large proportion of experts do not provide well calibrated assessments. An analysis on 322 experts [13] showed that only 27% of the experts’ assessments in 74 studies were calibrated, using a 0.05 threshold. By contrast, all experts in the three studies presented in this paper provided assessments leading to calibration scores smaller than 0.05. Finally, we emphasize that experts’ ability to provide valid assessments under uncertainty does not appear to correlate with years of experience or domain knowledge [18].

It is notable that the uncertainty in some attribution estimates is rather small, for example attribution of ETEC transmission to grains and beans in Ethiopia, or *Campylobacter* transmission to cooked red meat, also in Ethiopia. For these products, experts largely agreed on the attribution estimates. The uncertainty in other attribution estimates was higher, independent of a relatively low or high mean attribution estimate. For example, the expected attribution of milk from cattle due to *Campylobacter* was relatively high at 70%, and the 95% uncertainty interval ranged between 6% and 100%. On the other hand, the attribution estimates for offal due to *Salmonella* were rather low (36%), with an attendant large uncertainty interval ranging between 3% and 92%.

Attribution results for ETEC to food groups in Ethiopia and Burkina Faso were largely similar, even though only 2 experts provided estimates for both countries. Experts were explicitly instructed that there is no evidence that animal pathogenic ETEC strains are infectious to humans [19], but nevertheless in both countries, a sizeable proportion of ETEC illness was attributed to animal source foods. We asked the experts for their rationale behind this attribution, and they considered contamination of food products by infected food handlers can occur at any step in the food chain and may contribute to transmission of pathogens with human-only reservoirs by animal source foods. Likewise, attribution results for poultry meat and vegetables that were done in both countries were largely similar. A noteworthy exception is a higher attribution of poultry meat related illness to chicken meat consumed out of the home in Burkina Faso (~ 50%) vs. Ethiopia (~ 20%). This corresponds to a difference in the contribution of street food to chicken consumption in these countries [20,21].

To support decision making by national authorities, the attribution estimates presented in this study will be combined with national level estimates of the burden of foodborne disease from WHO FERG to present estimates of the burden of specific food groups and food products [10,11]. These data will inform priority setting of foods for control activities and can be combined with risk assessment and economic estimates to inform cost-benefit analyses.

## Supporting information

S1 Table Calibration questions used in the three African countries burden of foodborne disease source attribution expert elicitation. (DOCX)

S2 Table Detailed data for Figs 5–7 and Tables 3–7. (XLSX)
