# Supplementary material for: Attribution of country level foodborne disease to food group and food types in three African countries: Conclusions from a structured expert judgment study
Source: PLoS Negl Trop Dis. 2022 Sep 12;16(9):e0010663. doi: 10.1371/journal.pntd.0010663 (PMC9499278; doi:10.1371/journal.pntd.0010663)
Supplement: S1 Table — (DOCX) [file pntd.0010663.s001.docx]

**S1 Table. Calibration questions used in the three African countries burden of foodborne disease source attribution expert elicitation.**

| **Themes** | **Question** | **Lower credible value (5^th^ percentile)** | **Central value**  **(50^th^ percentile)** | **Upper credible value**  **(95^th^ percentile)** |
| --- | --- | --- | --- | --- |
| **Theme 1: The West Africa Ebola epidemic from 2013 to 2016.** | What was the total number of cases reported during the 2013-2016 Ebola outbreak from the three most affected countries Sierra Leone, Liberia and Guinea combined? |  |  |  |
|  | How many Ebola deaths occurred in these three most affected countries Sierra Leone, Liberia and Guinea combined in 2013 to 2016? |  |  |  |
|  | What is the overall number of current survivors of the 2013 to 2016 Ebola outbreak worldwide? |  |  |  |
|  | What was the number of participants in the Ebola vaccine trial (rVSV-ZEBOV) in Guinea during 2015? |  |  |  |
| **Theme 2: Diarrhea in Mali** | What is the percent of diarrheal deaths in all children under the age of five years old in Mali for 2012 to 2013? |  |  |  |
|  | In 2015, what was the incidence rate of diarrheal deaths in children under the age of five years old in Mali per 10,000 children? |  |  |  |
|  | What was the overall mortality rate due to unsafe WASH practices in Mali for year 2016 per 100,000 population? |  |  |  |
| **Theme 3: Food production in Uganda** | What is the average amount of animal proteins which a person used in Uganda in grams per day from 2005 to 2013? |  |  |  |
|  | What was the three-year average amount of total production of cereals in Uganda given in megatons from 2012 to 2015? |  |  |  |
|  | What was the percent of stunting in 2016 of all children under the age of five years old in Uganda? |  |  |  |
